# Some Injuries to the Vertebral Column

**Published:** 1909-03-06

**Authors:** 


### Surgery.

### SOME INJURIES TO THE VERTEBRAL COLUMN.

Injuries to the vertebral column may be divided for practical purposes into two classes—(*a*) those in which partial or complete recovery is spontaneous without operation, and (*b*) those in which definite pressure is produced upon the cord. The latter class may be again subdivided, according to the results of surgical interference, which may either be brilliantly successful, leading to total restitution of function, or equally disappointing, the patient not being benefited at all by operation.

The lesser injuries, in which spontaneous recovery occurs, are nearly always cases of concussion of the spinal cord, though the lesion is far from being common—surprisingly uncommon, in fact, when one remembers how frequent is concussion of the brain. Like the latter, it consists of a general disturbance of the spinal cord without any apparent lesion; it often escapes recognition, however, because the symptoms are somewhat vague; and it is difficult to give a concise account of its features. It is distinguished by abolition of motor and sensory function in all parts supplied from below the lesion, and by incontinence. In the course of a few hours the symptoms begin to pass off. A blacksmith while bending down in his forge was suddenly kicked in the gluteal region by a horse with sufficient violence to precipitate him against the opposite wall—a considerable distance. When first seen, he presented the symptoms of a total transverse lesion. These began to clear up in 24 hours, but it was some time before recovery was complete, and in the later stages of convalescence his symptoms were so anomalous and were so little compatible with a definite lesion that anyone seeing him then for the first time would have been justified in regarding them as being of functional origin.

If any constituent of the column is broken or displaced, or both broken and displaced at the same time, the outlook is very grave indeed. These injuries are called in text-books "fracture-dislocations of the spine," and it is said that a pure dislocation can only occur in the cervical region, the interlocking of the articular facets preventing this in other parts of the vertebral column. This is what one would expect. But in addition to this any or all of the processes of a vertebra may be broken and driven into the spinal cord, producing symptoms indistinguishable from the more severe lesion.

Symptoms of pressure may be produced, therefore, by a squeezing of the whole cord in a fracture-dislocation, or by a depressed fragment of a single vertebra, or by blood or callus. In the first two cases the symptoms follow immediately on the accident; blood-clot will not be collected in sufficient quantity to produce symptoms, at any rate for some hours, while callus, of course, will not make its presence known for some days.

In a complete transverse lesion of the cord there is at once complete paralysis of the flaccid type in the muscles supplied by the nerves which derive their origin from the anterior horn cells below the lesion, and there is complete anæsthesia up to the same level. All reflexes are abolished, and there is also incontinence of urine and fæces. This is not what one would expect on physiological grounds; for if the cord be divided the anterior horn cells in the lower half are presumably, for a time at any rate, undamaged and the nerves are in contact with their trophic centres, their connection with the higher or inhibitory centres alone being interfered with. One might reasonably suppose therefore that the paraplegia would be spastic in type, with exaggerated reflexes. It is difficult to explain why this does not occur. It might be argued that immediately after the accident the whole spinal cord is in a state of concussion. But then the reflexes should return in a few hours when the effects of the concussion pass off.

The prognosis varies with the level of the lesion. Roughly, it may be said that if the lesion is in the lower cervical region death occurs in a few days, in the dorsal region in a few weeks, and in the lumbar in a few months. In the latter case this is usually the result of an ascending pyelonephritis, although the greatest care may be taken to catheterise the bladder aseptically. A lesion at or above the level of the fourth cervical vertebra is incompatible with life, since the nerve supply to the diaphragm is cut.

Much help in localising the lesion may be obtained from a good skiagram, and this is particularly the case in the cervical region, when the photograph can be taken to display the lateral aspect.

It is often difficult to decide on the correct line of treatment. In the case of a dislocation in the cervical region, it seems reasonable to attempt reduction under anæsthesia by means of strong traction and manipulation. But this fails more often than not, because the displaced vertebra is tightly bound down in its abnormal position by surrounding structures, particularly the stretched ligamenta subflava, so that no traction is sufficient to reduce it. In the dorsal and lumbar regions it is hopeless even to attempt it.

In this case an operation should be performed, and the vertebral column thoroughly exposed at the level of the lesion. The next step will depend upon what is found. If the pressure is due to depressed lamina or laminæ, it is sufficient merely to remove them. In one case in the writer's knowledge a patient suffered from paraplegia as the result of an injury to his vertebral column for some months. On exposing the damaged area it was found that the laminæ of two of the lower dorsal vertebræ had been broken off and pressed up under the lamina of the vertebra above: the ligamenta subflava were intact. The depressed laminæ were removed, and the patient made a complete recovery. Even if there is no obvious irregularity of the spine viewed from behind, it is well to perform a laminectomy, for this will at least relive any pressure, and some restoration of function, even if incomplete, may follow. Some surgeons think it is not right to operate on these patients; but their condition cannot be made more wretched, and any proceeding which holds out the least hope of amelioration seems therefore justifiable.

